# Carbohydrate, glutathione, and polyamine metabolism are central to *Aspergillus flavus* oxidative stress responses over time

**DOI:** 10.1186/s12866-019-1580-x

**Published:** 2019-09-05

**Authors:** Jake C. Fountain, Liming Yang, Manish K. Pandey, Prasad Bajaj, Danny Alexander, Sixue Chen, Robert C. Kemerait, Rajeev K. Varshney, Baozhu Guo

**Affiliations:** 1 USDA-ARS, Crop Protection and Management Research Unit, Tifton, GA 31793 USA; 2 Department of Plant Pathology, University of Georgia, Tifton, GA 31793 USA; 3 College of Biology and Environmental Science, Nanjing Forestry University, Nanjing, 210037 China; 4 International Crop Research Institute for the Semi-Arid Tropics (ICRISAT), Hyderabad, Telangana 502324 India; 5 Metabolon, Inc., Durham, NC 27713 USA; 6 Department of Biology, Genetics Institute, and Plant Molecular & Cellular Biology Program, University of Florida, Gainesville, FL 32611 USA

**Keywords:** *Aspergillus flavus*, Aflatoxin, Drought stress, Oxidative stress, Metabolomics

## Abstract

**Background:**

The primary and secondary metabolites of fungi are critical for adaptation to environmental stresses, host pathogenicity, competition with other microbes, and reproductive fitness. Drought-derived reactive oxygen species (ROS) have been shown to stimulate aflatoxin production and regulate in *Aspergillus flavus*, and may function in signaling with host plants. Here, we have performed global, untargeted metabolomics to better understand the role of aflatoxin production in oxidative stress responses, and also explore isolate-specific oxidative stress responses over time.

**Results:**

Two field isolates of *A. flavus*, AF13 and NRRL3357, possessing high and moderate aflatoxin production, respectively, were cultured in medium with and without supplementation with 15 mM H_2_O_2_, and mycelia were collected following 4 and 7 days in culture for global metabolomics. Overall, 389 compounds were described in the analysis which encompassed 9 biological super-pathways and 47 sub-pathways. These metabolites were examined for differential accumulation. Significant differences were observed in both isolates in response to oxidative stress and when comparing sampling time points.

**Conclusions:**

The moderately high aflatoxin-producing isolate, NRRL3357, showed extensive stimulation of antioxidant mechanisms and pathways including polyamines metabolism, glutathione metabolism, TCA cycle, and lipid metabolism while the highly aflatoxigenic isolate, AF13, showed a less vigorous response to stress. Carbohydrate pathway levels also imply that carbohydrate repression and starvation may influence metabolite accumulation at the later timepoint. Higher conidial oxidative stress tolerance and antioxidant capacity in AF13 compared to NRRL3357, inferred from their metabolomic profiles and growth curves over time, may be connected to aflatoxin production capability and aflatoxin-related antioxidant accumulation. The coincidence of several of the detected metabolites in H_2_O_2_-stressed *A. flavus* and drought-stressed hosts also suggests their potential role in the interaction between these organisms and their use as markers/targets to enhance host resistance through biomarker selection or genetic engineering.

**Electronic supplementary material:**

The online version of this article (10.1186/s12866-019-1580-x) contains supplementary material, which is available to authorized users.

## Background

Abiotic stresses such as drought, heat, and osmotic stress have significant effects on the growth of plant pathogenic fungi, and can hinder their capability of infecting host plants. Drought stress in particular has been shown to have significant effects on both fungal pathogenicity and on host resistance to infection with some degree of specificity. For example, the growth of pathogenic fungi such as *Botrytis cinerea* causing gray mold, and *Oidium neolycopersici* causing powdery mildew on tomato are reduced or inhibited under drought stress [1, 2]. Drought can also influence host metabolic composition and affect interactions with invading pathogens [3]. The growth of microbes in soil environments along with their metabolic profiles and development can also be influenced by drought stress resulting in altered soil ecology and competition among microbes for limiting resources [4]. This shows the importance of metabolite accumulation in fungal environmental stress responses and pathogenicity. These observations also show the importance of host physiological and metabolic responses to abiotic stresses in regulating resistance to pathogen infection.

Members of the genus *Aspergillus* have been extensively studied using focused and untargeted metabolomics studies given their role as saprophytes in soil environments, their industrial applications, and their potential as human, animal, and plant pathogens. Examination of the metabolic responses of these fungi have been primarily focused on identifying metabolites involved in fungal growth and development, and the discovery of novel secondary metabolites encoded by silent, conserved gene clusters through both genomic prediction, and induced production using applied stressors or epigenetic modifying compounds [5–8]). This has led to the identification of a number of metabolites with potential pharmaceutical applications, and several involved in the regulation of fungal biology [9–11]. However, application of these techniques to study plant pathogenic species of *Aspergillus* have been limited.

An example of this is *Aspergillus flavus*, a facultative pathogen affecting crops such as maize and peanut which produces carcinogenic secondary metabolites termed aflatoxins. Annual losses can exceed $1 billion for US growers, particularly in regions susceptible to drought stress which has been shown to exacerbate aflatoxin contamination [12–14]. Recent examination of developing maize kernels under drought stress also showed that accumulation of polyunsaturated fatty acids, and simple sugars along with decreases in antioxidants such as polyamines occurred in inbred lines sensitive to drought stress and susceptible to aflatoxin contamination [15]. The same study also showed a greater accumulation of reactive oxygen species (ROS), specifically hydrogen peroxide (H_2_O_2_), in kernels of the drought sensitive line compared to the drought tolerant line under drought suggesting a correlation between drought tolerance and both ROS accumulation and aflatoxin contamination. Therefore, we hypothesize that investigating this correlation between both matrix composition and ROS accumulation with aflatoxin production in *A. flavus* may provide insights into mitigating drought-induced contamination.

Matrix composition has been shown to heavily influence both *A. flavus* growth and aflatoxin production. For example, carbon sources have been found to have a significant effect on aflatoxin production in vitro with simple sugars being able to support aflatoxin production by *A. flavus* and *A. parasiticus* while other carbon sources such as peptone can inhibit aflatoxin production in a concentration-dependent manner [16, 17]. Carbon source and availability has also been shown to influence conidiation in *A. flavus* [16]. In addition, the accumulation of lipid compounds such as unsaturated fatty acids, and oxylipins in host tissues have been demonstrated to influence aflatoxin production [18–21].

During in vitro experiments, the same ROS detected by Yang et al. [15] to accumulate in maize kernels under drought have also been shown to stimulate the production of aflatoxin in both *A. flavus* and *A. parasiticus*, and aflatoxin precursors in *A. nidulans* [22–25]. Variation in oxidative stress tolerance has also been observed among field and mutant isolates of *A. flavus* with isolates exhibiting greater aflatoxin production and more later-stage precursor production tending to tolerate greater levels of oxidative stress compared to less toxigenic or atoxigenic ones [16, 26]. Such variation in stress tolerance and growth patterns may also be characteristic of differences in isolate vegetative compatibility groups (VCGs) or mating-type locus-linked traits which have been shown to vary in host pathogenicity, and competitive ability with other isolates for environmental nutrients [27, 28]. Also, ROS function in reproductive signaling in *Aspergillus spp.* with oxidative responses being closely interconnected with the regulation of reproductive development [29]. Further, oxidative stress results in extensive metabolic profile alterations to fungi with regards to primary metabolism and antioxidant mechanisms following induction by either ROS or ROS-generating compound application [30–32].

Previous experimentation examining the oxidative stress responses of field isolates of *A. flavus* with different levels of aflatoxin production and stress tolerance by our group have shown a high degree of variability among isolates in overall strategies to remediate stress at both the transcript and protein levels [33–35]. These studies suggested that highly toxigenic isolates may exhibit earlier, more effective oxidative stress remediation mechanisms compared to less toxigenic or atoxigenic isolates. Transcripts and proteins involved in antioxidant protection, carbohydrate metabolism, microbial competitiveness, reproductive development, and the production of other secondary metabolites such as kojic acid and aflatrem were among those differentially expressed in response to oxidative stress. Differences in isolate-specific oxidative stress responses were also proposed to be due to resource allocation and the regulation of primary and secondary metabolic pathways to mitigate oxidative damage. While these studies provided an extensive overview of transcript and protein-level responses to oxidative stress, they are not fully capable of characterizing changes in final biochemical product levels, and resource allocation over time. Therefore, the objectives of this study were: 1) to identify differentially accumulating metabolites over time to explain isolate-to-isolate variability in oxidative stress responses; 2) to identify metabolic responses that begin to explain the relationship between oxidative stress and exacerbated aflatoxin production; and 3) to identify the metabolites that correspond to host drought responses with potential use in improving host resistance through selection or biotechnology. To accomplish this, we performed a global, untargeted metabolomics analysis of two field isolates of *A. flavus* with different levels of aflatoxin production and their response to oxidative stress over time.

## Results

### Effects of oxidative stress on isolate growth rates

Two isolates of *A. flavus*, AF13 and NRRL3357, which were previously observed to possess relatively high (up to 35 mM H_2_O_2_) and moderate (up to 20 mM H_2_O_2_) levels of oxidative stress tolerance and aflatoxin production, respectively [16], were selected for this study. The isolate AF13 is a high aflatoxin producing L-strain (sclerotia size > 400 μm) with a MAT1–2 mating type belonging to the YV-13 vegetative compatibility group (VCG) and relatively high tolerance to oxidative stress [16, 36, 37]. The isolate NRRL3357 is a moderately high aflatoxin producing L-strain with a MAT 1–1 mating type with no currently defined VCG, and moderate tolerance to oxidative stress [16, 38]. Using thin layer chromatography (TLC) (Additional file 1: Figure S1), it was found that aflatoxin B_1_ production was higher in AF13 than in NRRL3357 in the absence of oxidative stress at 7 DAI, but were more similar at 4DAI. Under stress aflatoxin B_1_ levels were increased in NRRL3357 and slightly decreased in AF13 at 7DAI, but were reduced in both isolates under stress at 4 DAI. Aflatoxin B_2_ levels were less than those for B_1_ as expected, showed similar patterns of accumulation under stress as seen for B_1_, and accumulated more in AF13 than in NRRL3357.

**Fig. 1:**
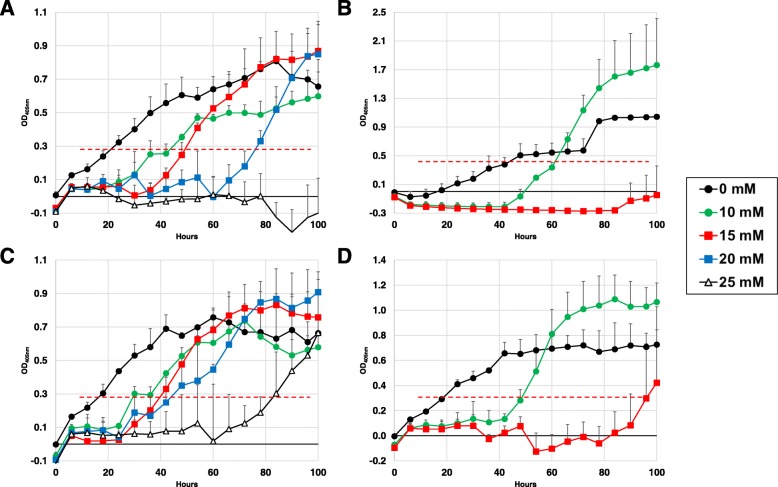
Growth curve analysis of *Aspergillus flavus* isolates AF13 and NRRL3357 under increasing oxidative stress and conidial concentration. The growth of AF13 (**a**, **c**) and NRRL3357 (**b**, **d**) were examined under increasing H_2_O_2_ concentrations in YES medium inoculated with either 2.0 × 10^4^ (**a**, **b**) or 8.0 × 10^4^ conidia/mL (**c**, **d**) by monitoring absorbance at 405 nm over 100 h. A threshold of 0.2 was selected for growth initiation timing which corresponded with linear phase initiation for both isolates in most conditions and is indicated by the dashed red line. Error bars represent standard deviation. No growth was detected at H_2_O_2_ concentrations > 15 mM in NRRL3357 with earlier growth initiation detected at higher conidia concentrations

These isolates were examined for conidial oxidative stress tolerance and the effect of oxidative stress on growth rates. Increasing levels of stress caused significant delays in the initial detection of isolate growth, interpreted as prolonged lag phase growth and delay of log phase initiation, for both isolates, but to a greater extent in NRRL3357 compared to AF13 at both inoculum levels (Fig. 1, Table 2). NRRL3357 showed numerically higher maximum growth rates than AF13 under lower levels of stress (Additional file 2: Table S1), but did not exhibit significant differences in maximum growth levels represented by maximum optical density (OD) nor in the time frame these maximum levels were attained (Additional file 3: Table S2). For AF13, significant growth delays were observed beginning at 10 mM H_2_O_2_ and increasing up to 25 mM where growth was completely suppressed at 20,000 conidia/mL (Fig. 1a) but not at 80,000 conidia/mL (Fig. 1c). However, growth was completely inhibited at 30 mM H_2_O_2_ even at the higher inoculum concentration (Table 1). For NRRL3357, significant delays in growth were also observed at 10 mM H_2_O_2_ while 15 mM was completely inhibitory of growth at 20,000 conidia/mL (Fig. 1b) but not at 80,000 conidia/mL (Fig. 1d). Growth was also completely inhibited at 20 mM H_2_O_2_ at the higher inoculum concentration (Table 1). These inhibitory concentrations of H_2_O_2_ observed for each isolate were 5 – 10 mM less than observed when the isolates were cultured in H_2_O_2_ amended YES medium in Erlenmeyer flasks with cotton plugs [16].

**Table 1:** Average detection times (OD_405_ = 0.2) during the growth curve assay

Isolate	Conidial Conc. (c/mL)	Detection Time by Treatment					
		0 mM	10 mM	15 mM	20 mM	25 mM	30 mM
AF13	20,000	14.42 ± 2.25 ghi	31.83 ± 3.54 efg	46.42 ± 2.50 cde	60.67 ± 13.32 bc	ND	ND
	80,000	9.42 ± 1.53 i	27.50 ± 1.09 f-i	36.82 ± 4.06 def	30.42 ± 6.15e-h	77.75 ± 9.59 ab	ND
NRRL3357	20,000	30.50 ± 8.40 e-h	54.92 ± 1.18 cd	ND	ND	ND	ND
	80,000	12.50 ± 0.87 hi	36.75 ± 8.55 def	88.38 ± 3.71 a	ND	ND	ND

*ND* Not Detected; Values with the same letters are not significantly different by Tukey’s Post Hoc (α = 0.05)

### Differential metabolic alterations in response to oxidative stress over time

Two field isolates of *A. flavus* were selected for global, untargeted metabolomics analysis using an UPLC-MS/MS approach to examine their responses to drought-related, H_2_O_2_-derived oxidative stress over time. This metabolomics analysis identified 389 distinct metabolites. Functional classification for the detected metabolites was performed based on the Kyoto Encyclopedia of Genes and Genomes (KEGG) database [39]. These metabolites were grouped into nine super pathways with a majority of metabolites being classified as either amino acids (163), lipids (84), nucleotides (54), or carbohydrates (43). These super pathways were further divided into 47 sub-pathways which are described in Additional file 4: Table S3.

Welch’s two-sample t-test was used for differential accumulation analyses to identify metabolites significantly different between oxidative stress treatments, between isolates, or over time (Table 2). Data normalization using DNA or protein content was found to introduce possible skewing in time and isolate effects on metabolite accumulation. Therefore sample mass per unit volume of extraction solvent was used for normalization and these data were used for analysis and interpretation. Both protein and non-protein normalized datasets, and raw data are included in Additional file 5. When comparing between stress treatments, AF13 showed 111 and 47 metabolites which differentially accumulated at 4 and 7 DAI, respectively. Of these, 27 and 64 metabolites were significantly increased and decreased, respectively, in abundance at 4 DAI, and 34 and 13 were increased and decreased in abundance, respectively, at 7 DAI. For NRRL3357, 223 and 90 metabolites were differentially accumulated at 4 and 7 DAI, respectively, in response to stress. Of these, 90 and 133 were significantly increased and decreased, respectively, at 4 DAI, and 65 and 25 were increased and decreased in abundance, respectively, at 7 DAI. Time was a highly significant influence on metabolite accumulation with AF13 showing 257 metabolites with significant differences in abundance between 4 and 7 DAI without H_2_O_2_treatment and 268 with H_2_O_2_ treatment. In NRRL3357, this was also apparent with 243 and 261 metabolites being significantly altered in abundance between 4 and 7 DAI either with or without H_2_O_2_ treatment, respectively. Comparisons between the isolates are more likely to reflect genetic differences rather than stress response, but more stark differences in numbers of differentially accumulating metabolites could be observed between AF13 and NRRL3357 at 4 DAI regardless of H_2_O_2_ treatment.

**Table 2:** Numbers of significantly, differentially accumulating metabolites

Effects	Comparison	Total Sig. Met.	Sig. Increased	Sig. Decreased
Time	A7N / A4N	257	58	199
	A7Y / A4Y	268	108	160
	N7N / N4N	243	51	192
	N7Y / N4Y	261	118	143
Stress	A4Y / A4N	111	27	84
	A7Y / A7N	47	34	13
	N4Y / N4N	223	90	133
	N7Y / N7N	90	65	25
Isolate	A4N / N4N	143	95	48
	A7N / N7N	97	50	47
	A4Y / N4Y	220	128	92
	A7Y / N7Y	96	42	54

Comparison nomenclature: AF13 (A), NRRL3357 (N), DAI (4 and 7), without H_2_O_2_ (N), and with H_2_O_2_ (Y)

**Fig. 2:**
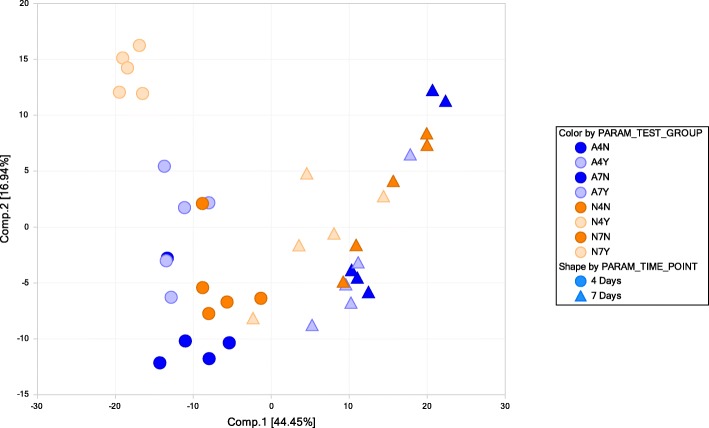
Principal components analysis (PCA) of metabolite accumulation. A4N, A4Y, A7N, and A7Y refer to AF13 at 4 and 7 DAI with and without 15 mM H_2_O_2_ treatment. N4N, N4Y, N7N, and N7Y refer to the same for NRRL3357. Dark blue points correspond with AF13 with no stress and light blue points refer to AF13 with stress. Orange points correspond with NRRL3357 with no stress and light orange points refer to NRRL3357 with stress. Circles represent samples at 4 DAI and triangles represent samples at 7 DAI

These differences in metabolite accumulation were also observed in principal components analyses (Fig. 2). The first component was dominated primarily by time effects reflecting significant differences between the 4 and 7 DAI time points. Significant stress effects could also be observed between the isolates with more stark differences observed at 4 DAI. Samples from 7 DAI did not segregate into distinct clusters as seen in samples from 4 DAI. A higher degree of variability between biological replicates was also observed in the 7 DAI samples compared to 4 DAI.

### Carbohydrate metabolic responses to oxidative stress

**Fig. 3:**
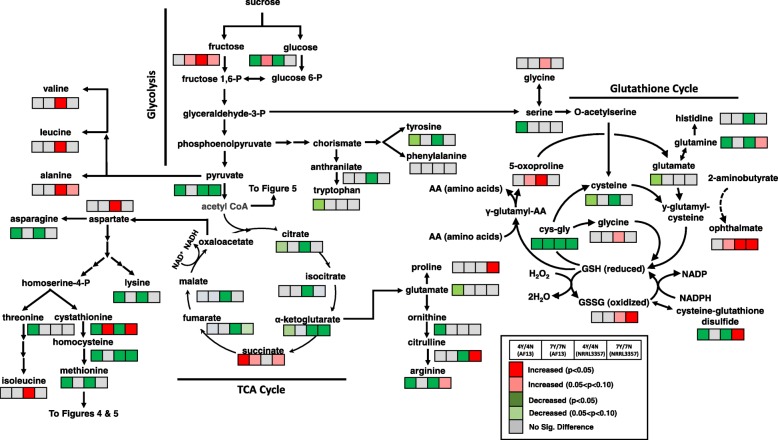
Differential accumulation of compounds involved in carbohydrate metabolism, glutathione metabolism, and amino acid biosynthesis. Heatmaps located at each metabolite represent the changes in metabolite accumulation in response to oxidative stress in AF13 and NRRL3357 at 4 and 7 DAI. Red and green indicate significant increases and decreases in metabolite levels, respectively (*p* < 0.05). Light red and light green indicate marginally significant increases and decreases in metabolite levels, respectively (0.05 < *p* < 0.10). Grey represents no significant changes

Significant variation in carbohydrate metabolite accumulation was observed in response to oxidative stress in both AF13 and NRRL3357. AF13 showed significant changes in glycolytic compounds glucose and pyruvate with significant decreases (*p* < 0.05) in both compounds at 4 DAI in response to stress with glucose and fructose levels showing marginally significant increases at 7 DAI (*p* < 0.10; Fig. 3). NRRL3357 showed significant decreases in both glucose and pyruvate at 4 DAI in response to stress with a significant decrease in pyruvate also detected at 7 DAI. Fructose levels in NRRL3357 were also increased at both time points in response to stress (Fig. 3). Time effects showed that pyruvate accumulated in NRRL3357 over time regardless of H_2_O_2_ treatment, and glucose and fructose were significantly decreased over time with reductions in glucose only seen in non-stressed samples (Additional file 5).

Intermediates in the tricarboxylic acid (TCA) cycle were also significantly affected by oxidative stress. NRRL3357 showed significant reductions, particularly at 4 DAI, in citrate, isocitrate, alpha-ketoglutarate, fumarate, and malate in response to oxidative stress (Fig. 3). Conversely, AF13 showed no significant changes in TCA intermediate levels in response to oxidative stress with the exception of a significant increase in succinate at 4 DAI. These compounds were, however, seen to generally accumulate over time in the stressed samples when comparing time points (Additional file 5).

In addition to these pathways, AF13 and NRRL3357 showed significant reductions in trehalose, arabitol, and xylitol in response to oxidative stress at 4 DAI with less significant decreases or no significant differences being observed in response to stress at 7 DAI (Additional file 5). Additional metabolic products of arabinose and xylinose, arabinate and xylinate were increased in accumulation in response to stress in both isolates and time points (Additional file 5). Increases in amino sugars were also observed in both isolates, particularly at 4 DAI in response to stress (Additional file 5).

### Amino acid metabolic responses to oxidative stress

Significant changes in the accumulation of amino acids and their derivatives were observed in both isolates in response to oxidative stress over time. Changes in primary amino acids were proportional to changes in their precursors with more significant changes occurring in NRRL3357 compared to AF13 (Fig. 3; Additional file 5). In particular, changes in aromatic amino acid precursors in the tryptophan and histidine pathways were observed in NRRL3357 in response to oxidative stress although the levels of tryptophan and histidine were unchanged or reduced, respectively, in the same conditions (Additional file 5). In addition, the tryptophan derivative kynurenine was increased in NRRL3357 at both time points in response to oxidative stress, but not in AF13 (Additional file 5). Proline levels were also increased in NRRL3357 at 7 DAI in response to stress (Additional file 5). Among the amino acid derivatives, those involved in glutathione, polyamine, and sulfur metabolism were among the most differentially accumulating in response to oxidative stress.

Glutathione metabolism was significantly regulated in both isolates but to a greater extent in NRRL3357 compared to AF13 (Fig. 3). Significant increases in 5-oxoproline, ophtalmate, oxidized glutathione (GSSH), and cysteine-glutathione disulfide were observed in NRRL3357 in response to increasing stress (Fig. 3). AF13 showed marginally significant (*p* < 0.10) increases in accumulation of only 5-oxoproline and ophtalmate were see at 7 DAI in response to stress. Direct comparison of levels between these isolates showed that AF13 accumulated significantly greater levels of GSSH and cysteine-glutathione disulfide at 4 DAI compared to NRRL3357 in the absence of oxidative stress, and equivalent and greater levels, respectively, of each when under oxidative stress (Additional file 5). When comparing time point measurements, 5-oxoproline, ophtalmate, and GSSG showed significant reductions in accumulation in both isolates and treatments (Additional file 5). Significant changes were also found among the gamma-glutamyl amino acids which were significantly reduced in AF13 at 4 DAI in response to stress, but tended to be either unchanged or increased in accumulation in NRRL3357 in response to stress (Fig. 3).

**Fig. 4:**
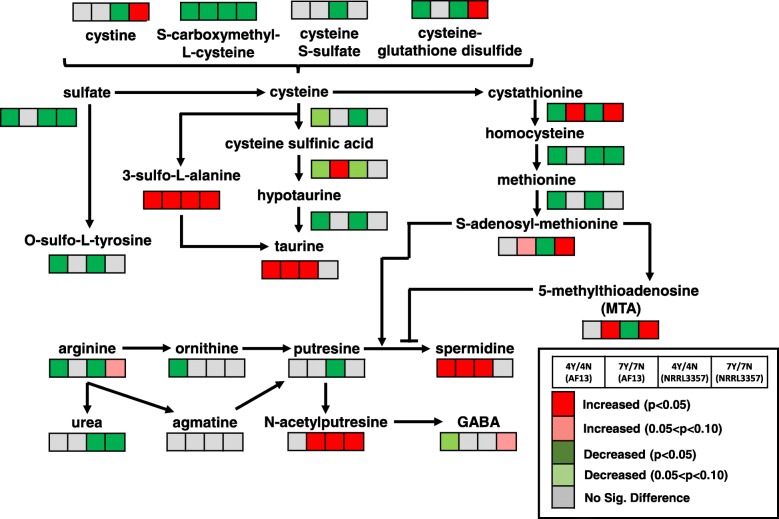
Differential accumulation of compounds involved in polyamine and sulfur metabolism. Heatmaps located at each metabolite represent the changes in metabolite accumulation in response to oxidative stress in AF13 and NRRL3357 at 4 and 7 DAI. Red and green indicate significant increases and decreases in metabolite levels, respectively (*p* < 0.05). Light red and light green indicate marginally significant increases and decreases in metabolite levels, respectively (0.05 < *p* < 0.10). Grey represents no significant changes

In addition to glutathione, other sulfur-containing amino acids and their metabolites were significantly regulated in response to oxidative stress (Fig. 4). Significant reductions in methionine levels were observed in both isolates at 4 DAI in response to oxidative stress. S-adenosylmethionine (SAM), an important signaling compound, was also significantly regulated in response to oxidative stress showing increasing accumulation at 7 DAI in both isolates, and a significant decrease at 4 DAI in NRRL3357 (Fig. 4). 5-methylthioadenesine (MTA) also exhibited a similar pattern of accumulation to SAM. In addition to methionine derivatives, cysteine also serves as a precursor to the antioxidant compound taurine which was significantly increased in both isolates at 4 DAI and in AF13 at 7 DAI in response to oxidative stress. A taurine precursor, 3-sulfo-L-alanine, was also significantly increased in both isolates and time points in response to oxidative stress (Fig. 4).

Polyamine metabolites were also significantly regulated in response to oxidative stress in both isolates. Ornithine showed significant reduction in AF13 at 4DAI while putresine showed the same in NRRL3357 in response to oxidative stress while the immediate precursor to ornithine, N-alpha-acetylornithine, was increased in both isolates at 4 DAI (Fig. 4; Additional file 5). These compounds are precursors to both spermidine and N-acetylputresine which showed significant increases in both isolates in response to oxidative stress. N-acetylputresine is a part of butanoate metabolism and used for the biosynthesis of gamma-aminobutanoate (GABA) which showed marginally significant changes in abundance in response to oxidative stress (Fig. 4).

### Fatty acid metabolic responses to oxidative stress

**Fig. 5:**
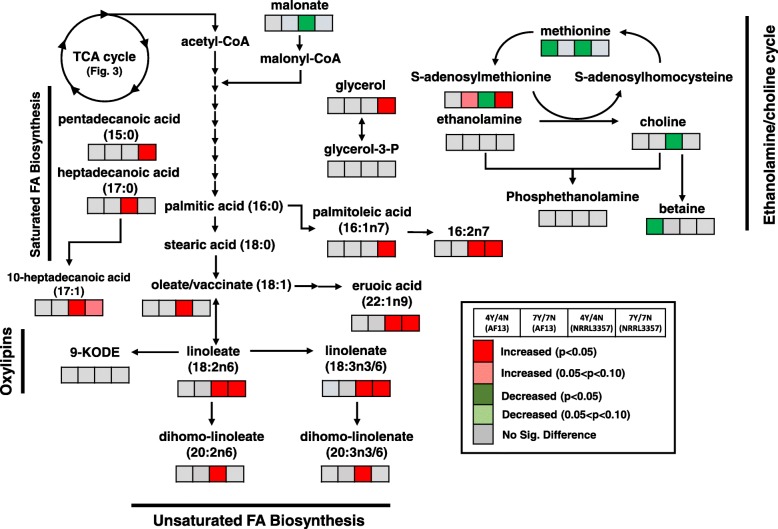
Differential accumulation of compounds involved in lipid metabolism. Heatmaps located at each metabolite represent the changes in metabolite accumulation in response to oxidative stress in AF13 and NRRL3357 at 4 and 7 DAI. Red and green indicate significant increases and decreases in metabolite levels, respectively (*p* < 0.05). Light red and light green indicate marginally significant increases and decreases in metabolite levels, respectively (0.05 < *p* < 0.10). Grey represents no significant changes

Several fatty acids and their derivatives were also significantly regulated in response to H_2_O_2_-stress over time. Significant regulation of saturated and mono- and poly-unsaturated fatty acid accumulation were primarily observed in NRRL3357 in response to stress (Fig. 5). Significant increases in the saturated fatty acids pentadecanoic acid (15:0) and heptadecanoic acid (17:0) were seen at 7 and 4 DAI, respectively, in NRRL3357 in response to stress (Fig. 5). Similarly, significant increases in several unsaturated fatty acids were also seen in NRRL3357 (Fig. 5). While no significant regulation of these fatty acids was seen in AF13 in response to oxidative stress within each time point, significant depletion of these fatty acids was observed in both isolates over time with or without the presence of oxidative stress (Additional file 5).

Other fatty acid derivatives were also found to differentially accumulate in the isolates under oxidative stress. In AF13, betaine, an ethanolamine derivative, was significantly decreased under oxidative stress at 4 DAI (Fig. 5). Several phospholipids such as glycerophosphoglycerol were also found to be differentially accumulating in response to oxidative stress in both isolates (Additional file 5). Ergosterol levels were found to be significantly decreased under stress in AF13 at 7 DAI and in NRRL3357 at 4 DAI. There was no significant change in ergosterol levels over time in either treatment, but AF13 accumulated significantly more than NRRL3357 at 4 DAI in both treatments, while at 7 DAI AF13 was found to have more only in the non-stressed control (Additional file 5).

### Other compounds regulated in response to oxidative stress

In addition to amino acids, carbohydrates, and lipids, other classes of compounds were found to differentially accumulate in response to increasing oxidative stress over time in both isolates. Among cofactors and electron carriers, carnitine and related metabolites were significantly reduced in both isolates at 4 DAI in response to stress, but showed significant increases over time under stress and was present in higher concentrations in AF13 compared to NRRL3357 (Additional file 5). Several B vitamins with potential antioxidant activity were also regulated in response to stress including thiamin (B1), riboflavin (B2), and pyridoxine (B6) (Additional file 5). Several nucleotide derivatives were also differentially accumulated in response to stress such as adenosine 5′-monophosphate (AMP) which was significantly increased in AF13 at 4 DAI in response to stress, but was significantly depleted in both isolates and treatments over time (Additional file 5). Terpenoid and isoterpenoid precursors were also found to differentially accumulate under stress with mevalonate along with its immediate precursor, 3-hydroxy-3-methylglutarate, and its lactone form, mevalonolactone, showing significant increases in response to stress in AF13 at 7 DAI and in NRRL3357 at 4 and 7 DAI (Additional file 5).

## Discussion

Aflatoxin production capability has been previously correlated with isolate oxidative stress tolerance [16, 26]. When examining the growth rates and behavior of *A. flavus* isolates under oxidative stress AF13, a highly toxigenic isolate, was found to exhibit higher levels of oxidative stress tolerance and growth under stress compared to NRRL3357, a moderately high toxigenic isolate (Fig. 1). Aflatoxin production and the intermediate reactions in the biosynthetic pathway are suspected to result in increased conidial oxidative stress tolerance due to stimulating additional antioxidant enzyme production, or through the consumption of ROS during production [26, 33]. Given this, conidial antioxidant enzyme activity may have contributed here. To further examine this, growth curve analyses was performed with different inoculum concentrations, and showed that growth for both isolates occurred at elevated H_2_O_2_ levels when the inoculum was increased from 20,000 conidia/mL as described by Meletiadis et al. [40] to 80,000 conidia/mL used here as inoculum for cultures used for metabolomics analysis. Interestingly, even with the increased conidia concentration, observed stress tolerance remained approximately 5 mM less than the maximum observed in the previous study. This may be an artifact of performing the assay in a sealed microplate which likely limited oxygen availability which can reduce growth and possibly aflatoxin production in the assay. This possibility is also bolstered by the observation of sparse conidiation in wells along the border of the plates which may have received oxygen from leaks in the microplate seal (data not shown). Normal conidiation was observed when culturing the metabolomic cultures in flasks sealed with sterile cotton which allows gas exchange and points to oxygen deprivation as the cause of the discrepancy in maximum tolerance between the methods. Regardless, the overall trend was consistent with previous observations of each isolate’s tolerance to oxidative stress [16, 23, 41]. Increased tolerance and more growth under increasing stress in AF13 also points to a correlation between growth, and aflatoxin production and conidial catalase and/or other antioxidant capacity as described by Roze et al. [26]. Numerically higher levels of maximum changes in growth rates (Additional file 2: Table S1) in NRRL3357 are also likely due to differences in growth behavior between the isolates. For example, NRRL3357 tends to exhibit greater levels of aerial mycelial growth with less conidiation while AF13 shows very planar growth with high levels of conidiation, traits concurrent with the isolates’ mating types.

When examining overall metabolite accumulation patterns, NRRL3357 displayed approximately double the number of differentially accumulating metabolites compared to AF13 in response to oxidative stress with both isolates exhibiting greater numbers at 4DAI compared to 7 DAI (Table 2). This pattern mirrors observed numbers and functional classifications of differentially expressed transcripts and proteins for these isolates in response to similar levels of oxidative stress in our previous transcriptome and proteome studies [33–35]. Here, significant differences in metabolite accumulation were detected within and between time points in both isolates, and sampling time was one of the major grouping factors in the PCA analysis (Table 2; Fig. 2 and Additional file 1: Figure S2). These time course influences may be due to differences in isolate growth patterns and, presumably, timing and vigor of oxidative stress remediation mechanisms. As indicated by the growth curve analysis (Fig. 1; Table 1), earlier initiation of growth in AF13 compared to NRRL3357 may be the result of earlier, more vigorous lag phase or conidial oxidative stress remediation processes. Therefore, sampling at 4 DAI for both isolates would describe actively growing and responding tissues while sampling at 7 DAI would describe stationary state responses in AF13 having already remediated the majority of oxidative stress while NRRL3357 would still be actively growing and responding to stress. These delays in growth are also likely to be the cause of the reduced levels of aflatoxin found to be produced by both isolates with increasing stress at 4 DAI while increases could be seen at 7 DAI (Table 1, Additional file 2: Table S1, and Additional file 3: Table S2).

Examining the isolate-specific responses to oxidative stress, there were significant differences in carbohydrate accumulation. Both glycolysis and TCA cycle intermediates were significantly altered in accumulation in response to oxidative stress in these isolates, but to differing degrees. NRRL3357 displayed increased demand for TCA intermediates showing significant decreases in most quantified metabolites in the cycle while AF13 showed no significant differences (Fig. 3). These compounds have been shown to provide some antioxidant benefit when supplemented to cultured neuronal cells [42], though a more likely explanation is the use of these compounds in the synthesis of amino acids and/or their derivatives involved in oxidative stress remediation. Increases in glucose and fructose under stress in both isolates may also be reflective of higher levels of metabolic demand for simple sugars, and the beginnings of carbon starvation leading to gluconeogenesis [43, 44], particularly at 7 DAI (Fig. 3).

When examining time course effects, the accumulation of these compounds in stressed samples may also be indicative on increased energy demand and the need to maintain redox homeostasis through the generation of reduced coenzymes for oxidative phosphorylation such as NADH and NADPH [45]. Significant reductions in their accumulation in NRRL3357 under stress (Fig. 3) could, therefore, partially explain the reduced growth rate, and observed ongoing stress responses compared to AF13. Also of interest, the pentose phosphate pathway has been shown to be involved in oxidative stress responses in yeast, and amino sugars such as ribonate are also involved in the generation of reduced coenzymes used for redox homeostasis [46, 47]. These reduced coenzymes, particularly NADPH, are also critical for the activity of polyketide synthases which may also impact aflatoxin production levels under oxidative stress [48–50].

Changes in amino acid metabolite levels appeared to form the basis of a majority of the oxidative stress remediation processes employed by these isolates constituting the bulk of directly antioxidant compounds and mechanisms. Amino acids such as proline have been previously shown to be involved in osmotic, drought, and oxidative stress tolerance in fungi and plants and were increased in abundance in NRRL3357 [51, 52]. Also in NRRL3357, the tryptophan derivative kynurenine was increased and may also contribute to stress remediation. Disruption of kynurenine 3-monooxygenase, a central enzyme in kynurenine metabolism, in *Botrytis cinerea* has been shown to increase tolerance to H_2_O_2_-derived oxidative stress and host pathogenicity while negatively affecting growth and development [53].

Glutathione pathway components were among the most significantly altered in response to oxidative stress in both isolates, though to a greater extent in NRRL3357 which can be seen in the higher accumulation of oxidized glutathione, 5-oxoproline, and ophthalmate in NRRL3357 under stress which were not seen in AF13 (Fig. 3). This pathway in conjunction with enzymes such as catalases and thioredoxin reductases and peroxidases serve as the primary means of redox homeostasis and oxidative stress alleviation for eukaryotes [54]. Glutathione metabolism has been previously linked to both development and aflatoxin production in *Aspergillus spp*. Huang et al. [48] showed that treatment of *A. flavus* with an ethylene-producing compound resulted in increases in GSH/GSSH ratios, oxidative stress remediation, and significant reductions in aflatoxin biosynthetic gene expression and aflatoxin production. Reduced glutathione accumulation has also been associated with asexual and sexual development in *A. nidulans* thioredoxin A (*AnTrxA*) mutants with applied GSH resulting in restored conidiation and early induction of cleistothecia formation following long-term, low concentration application [55]. Given this relationship between glutathione, development, and mycotoxin production, this mechanism may be lending to distinctive growth patterns and aflatoxin production levels in these isolates which represent diverse VCGs and mating types and warrants further investigation [27, 28, 56].

Sulfur-containing amino acids such as cysteine and methionine, and their derivatives were also differentially accumulated in response to oxidative stress (Fig. 4). These compounds have antioxidant benefits, and also function in important signaling capacities. Taurine, an antioxidant compound [57], was shown to accumulate in both isolates under stress along with its immediate precursor 3-sulfo-L-alanine which may supplement other antioxidant pathways (Fig. 4). The detected signaling compounds, SAM and MTA, are closely tied to polyamine biosynthesis which was also significantly regulated by oxidative stress. Polyamines such as putresine and spermidine differentially accumulated in this experiment (Fig. 4), and have been found to function in oxidative stress responses either by scavenging ROS, inhibiting ROS-generating enzymes, or functioning in signal transduction to promote antioxidant mechanisms [58]. S-adenosylmethionine is required for the production of polyamines and MTA is produced from decarboxylated SAM by spermidine synthase and spermine synthase with accumulating MTA being able to inhibit these enzymes to prevent the generation of H_2_O_2_-derived oxidative stress due to polyamine back-conversion [59]. Therefore, polyamine metabolism along with glutathione metabolism form a coordinated basis for regulating cellular redox potential in *A. flavus* in response to oxidative stress and may assist in coordination of both reproductive development and mycotoxin production.

Fatty acids were also significantly altered in accumulation in response to oxidative stress. This is particularly true for mono- and poly-unsaturated fatty acids which tended to be increased in abundance in NRRL3357 in response to stress, but not in AF13 (Fig. 5). Unsaturated fatty acids have been found to be suitable substrates for aflatoxin production by *A. flavus* and *A. parasiticus*, and their byproducts have been shown to regulate aflatoxin production and development [60, 61]. For example, linoleic acid derivatives known as Psi factors have been shown to regulate both asexual and sexual sporulation in *A. flavus* and *A. nidulans* [62], and oxylipins function in signaling for development, mycotoxin production, and host interactions [21, 63, 64]. In addition to signaling, free fatty acids also serve as important sources of energy, and can be catabolized to produce other macromolecules. Here, a majority of unsaturated lipids were depleted over time in control and stressed conditions in both isolates likely to provide energy and components for repairing and responding to oxidative stress (Additional file 5). These fatty acids are also important for maintaining membrane integrity and fluidity under environmental stress conditions. For example, dienoic fatty acids have been shown to function in preserving membrane fluidity in yeast under freezing and salt stresses [65].

Along with these major classes of metabolites, several cofactors and secondary metabolites were also differentially accumulated in response to stress (Additional file 5). Of particular interest were mevalonate and related terpenoid compounds which were increased in both isolates in response to oxidative stress. These compounds are precursors to some isoprenoid mycotoxins such as aflatrem whose biosynthetic genes have been found to be upregulated in response to oxidative stress in these isolates [33, 34]. In addition, mevalonate and its derivatives have been shown to link the biosynthetic pathways for ergosterol and ornithine-derived siderophores, and interruption of this link results in reduced tolerance to oxidative stress, siderophore production, and virulence in *A. fumigatus* [66].

These compounds differentially accumulating in these isolates of *A. flavus* mirror those observed in other *Aspergillus spp.* such as *A. oryzae* [67] and provide potential insights into putative approaches to enhance host resistance under drought stress. We hypothesized that excessive ROS generated in drought sensitive host plants during drought stress may contribute to enhancing susceptibility to aflatoxin contamination [16, 68]. In addition, the metabolic pathways employed by *A. flavus* in remediating oxidative stress seen in this study parallel those employed by host plants such as maize in countering drought stress in developing kernel tissues [15]. Given this relationship, the manipulation of host tissue composition may be a viable approach to improve aflatoxin contamination resistance through two possible methodologies. The first method is biomarker selection employed in breeding programs [69]. For aflatoxin mitigation, enhanced accumulation of antioxidant compounds in host plant tissues corresponding to those observed in *A. flavus* such as glutathione pathway components, polyamines, or simple sugar content could be selected for in conventional and molecular breeding programs. The second method is genetic engineering including both genome editing and transgenic approaches to manipulate the expression of host plant enzymes to modify kernel composition to reduce stress on infecting *A. flavus* under drought. These technologies could also be used to enhance host plant antioxidant potential through increased antioxidant enzyme expression, antioxidant compound production, or aflatoxin inhibitor production. This would also have the added potential benefit of reduced drought-related kernel abortion and filling reduction due to oxidative damage.

## Methods

### Isolate collection

The isolates used in this study were obtained as follows. AF13 was requested from Dr. Kenneth Damann, Department of Plant Pathology and Crop Physiology, Louisiana State University, Baton Rouge, LA. NRRL3357 was requested from the USDA National Culture Repository, Peoria, IL. All isolates were shipped on PDA and transferred to V8 agar as previously described [35]. Agar plugs containing fresh conidia were taken along the growing edge of the colonies and stored in sterile water and 20% (v/v) glycerol at 4 and − 20 °C, respectively, until used.

### Culture conditions and tissue collection

Isolate conidia suspensions were used to inoculate V8 agar plates, and were incubated at 37 °C for 5 days. Conidia were then harvested using sterile 0.1% (v/v) Tween 20 and a sterile loop to make a fresh conidia suspension (~ 2.0 × 10^7^ conidia/mL based on hemocytometer measurements) for use as inoculum. For each isolate, 100 μL of conidial suspension was then used to inoculate stationary liquid cultures of 50 mL yeast extract-sucrose medium (YES; 2% yeast extract, 1% sucrose) in 125 mL Erlenmeyer flasks amended with H_2_O_2_ (3% stabilized solution) to a final concentration of either 0 or 15 mM and a final conidia concentration of ~ 8.0 × 10^4^ conidia/mL. This concentration was selected based on previous studies on the responses of these isolates to oxidative stress in general [16], and in greater detail at the transcript [33, 34] and protein [35] levels. This concentration yielded significant responses to stress with a lesser degree of effects on isolate development in these studies. The flasks were plugged with sterile cotton and incubated at 30 °C in the dark. Mycelial mats were then harvested for each isolate and H_2_O_2_ treatment at 4 and 7 days after inoculation (DAI). Five repeat cultures representing five biological replicates were harvested for each isolate, treatment, and time point. A detailed description of the experiment design can be found in Additional file 1: Figure S2. Harvested mycelia mats were immediately flash frozen in liquid nitrogen and ground into a fine powder using sterile, chilled mortar and pestles. A portion of the ground tissue (~ 1 g) was then transferred to a sterile 2.0 mL microcentrifuge tube and stored at − 80 °C until used for the metabolomics analysis.

### Metabolomic profiling

Collected and ground mycelia tissues were used for global, unbiased metabolomics by Metabolon (Morrisville, NC, USA) as described by Yang et al. [15] and Lin et al. [70]. Briefly, 50 mg of tissue from each sample were prepared using an automated MicroLab STAR system (Hamilton, Reno, NV, USA) during which QC standards were added for downstream normalization. Metabolites and proteins were extracted in methanol in a GenoGrinder 2000 (Glen Mills, Clifton, NJ, USA) followed by centrifugation for metabolite isolation and protein separation. Each extract was then divided into 5 fractions and used for reverse phase (RP)/ultra-performance liquid chromatography (UPLC)-tandem mass spectrometry (MS/MS) with positive ion mode electrospray ionization (ESI), RP/UPLC-MS/MS with negative ion mode ESI, and HILIC/UPLC-MS/MS with negative ion mode ESI. One fraction from each extract was reserved as a backup. All methods employed either an ACQUITY UPLC (Waters, Milford, MA, USA) or a Q-Exactive High Resolution/Accuracy Mass Spectrometer with a heated electrospray ionization (HESI-II) source and an Orbitrap Mass Analyzer (ThermoFisher, Waltham, MA, USA). A detailed description of methods and procedure for data acquisition, metabolite acquisition, quantitation, and data analysis can be found in Additional file 6.

### Growth curve assay

A growth curve assay was performed for the isolates used for metabolomics analysis under H_2_O_2_-derived stress using a microtiter plate method as described by Meletiadis et al. [40]. Both isolates were cultured on V8 agar for 7 days at 30 °C in the dark. Agar plugs were collected along the growing edge of the colonies and placed into amber bottles containing ~ 5.0 mL 0.1% (v/v) Tween 20 and gently shaken to suspend conidia. The concentration of each conidial suspension was measured using a hemocytometer, and used to prepare inoculum for each isolate with at two concentrations of 2.0 × 10^4^ conidia/mL as described by Meletiadis et al. [40] and 8.0 × 10^4^ conidia/mL as used for the present metabolomics assay. A 96-well flat bottom microtiter plate was then prepared by filling each well with 100 μL of double strength YES medium (4% yeast extract, 2% sucrose) amended with 0, 20, 30, 40, 50, or 60 mM H_2_O_2_. For each inoculum, 100 μL was added to each the prepared wells resulting in a standard YES concentration and a final concentration of 0, 10, 15, 20, 25, or 30 mM H_2_O_2_. Three replicate wells were inoculated for each isolate and treatment combination. For non-inoculated wells, 100 μL of 0.1% Tween 20 was added in place of inoculum. The plate was sealed with optically-clear tape and incubated at 30 °C in the dark without shaking in a Synergy HT plate reader (Biotek, Winooski, VT, USA). Optical density at 405 nm (OD_405_) was recorded every 15 min for 100 h. The average of the OD_405_ for the non-inoculated wells was then subtracted from each measurement to remove background absorbance.

### Thin layer chromatography

Thin layer chromatography (TLC) was performed at previously described [16] with modifications. Culture medium (~ 5 mL) from samples used in the metabolomics analysis were filtered through a 0.2 μm filter and stored at 4 °C in amber vials until used. For aflatoxin extraction, 700 μL of medium was combined with an equal volume of methylene chloride in a 2 mL microfuge tube, and shaken with an orbital shaker for 1 h. The samples were then centrifuged at room temperature for 10 min, and the lower organic layer was pipetted into an amber glass vial and allowed to evaporate completely overnight in the fume hood. The extracted aflatoxins were then suspended in 70 μL methylene chloride (10x concentration), of which 5 μl of each sample were spotted onto a silica coated glass TLC plate (20 × 20 cm, 105,715, Millipore-Sigma, Burlington, MA, USA). The plate was then developed in 96:3:1 (v/v) diethyl ether:methanol:water and placed under UV light (365 nm) for qualitative aflatoxin visualization and evaluation.

### Data analysis

Raw data obtained from UPLC-MS/MS analyses were peak-identified and QC corrected based on the Metabolon Laboratory Information Management System (LIMS) which contains identifying information for > 4500 standard compounds. Quantitation and differential accumulation analyses were performed as described by Lawton et al. [71], Lin et al. [70], and Rao et al. [72] using ArrayStudio and R (v3.4.0). Heatmaps and principal components analyses were performed using MultiExperiment Viewer (MeV, v4.9.0). Functional enrichment analyses were performed with Blast2GO [73], and metabolic pathways were identified based on the KEGG database [39]. Pearson correlation analyses of the detected metabolites was performed using R (v3.4.0) and RStudio (v1.1.423). For the growth curve analysis, Gen3 software (Biotek) was then used to calculate the highest OD (OD_max_), and average time of initial detection at a defined threshold of OD_405_ = 0.2 (T_i_).

## Additional files

Additional file 1: **Figure S1.** Thin layer chromatograph (TLC) of aflatoxins from culture medium extracts. Culture medium from each isolate were filtered and saved for use in aflatoxin quantification. Following extraction, samples from each isolate and treatment were developed with a TLC. Aflatoxins B_1_ and B_2_ can be observed on the TLC. Treatments are indicated below for each isolate, stress condition, and timepoint (DAI: days after inoculation). Fluorescence of the aflatoxins is visualized here using ultraviolet light (365 nm). **Figure S2.** Metabolomics experiment design. Two isolates of *Aspergillus flavus*, AF13 (highly aflatoxigenic and oxidative stress tolerant) and NRRL3357 (moderate to highly aflatoxigenic and moderately oxidative stress tolerant), were grown in yeast extract sucrose (YES) medium supplemented with either 0 or 15 mM H_2_O_2_. Samples were collected at 4 and 7 days after inoculation (DAI). Five biological replicates (*n* = 5, *N* = 40) were performed for each isolate, treatment, and time point combination. Statistical comparisons are indicated by the colored arrows with blue indicating oxidative stress effect comparisons, red indicating time effects, and green indicating isolate/genotype effects. (DOCX 842 kb)

Additional file 2: **Table S1.** Maximum changes in average growth rates (ΔOD_405_/hr) observed during the growth curve assay. (XLSX 9 kb)

Additional file 3: **Table S2.** Maximum OD_405_ observed in each isolate and treatment during the growth curve assay. (XLSX 10 kb)

Additional file 4: **Table S3.** Super & sub-pathways identified by KEGG analysis of the detected metabolites. (XLSX 11 kb)

Additional file 5: Fold Changes in Metabolite Accumulation (XLSX 594 kb)

Additional file 6: Detailed Metabolomics Methodology (PDF 636 kb)

## Data Availability

Raw and analyzed data are provided in the attached supplementary files.

## Abbreviations

AMP: Adenosine-5′-Monophosphate
DAI: Days after Inoculation
ESI: Electrospray Ionization
GABA: Gamma-Aminobutanoate
GSH: Reduced Glutathione
GSSH: Oxidized Glutathione
HESI: Heated Electrospray Ionization
MTA: 5-Methylthioadenosine
OD: Optical Density
ROS: Reactive Oxygen Species
RP/UPLC-MS/MS: Reverse Phase/Ultra-Performance Liquid Chromatography-tandem Mass Spectrometry
SAM: S-Adenosylmethionine
TCA: Tricarboxylic Acid Cycle
VCG: Vegetative Compatibility Group
YES: Yeast Extract-Sucrose
