# Supplementary material for: Carbohydrate, glutathione, and polyamine metabolism are central to Aspergillus flavus oxidative stress responses over time
Source: BMC Microbiol. 2019 Sep 5;19:209. doi: 10.1186/s12866-019-1580-x (PMC6727485; doi:10.1186/s12866-019-1580-x)
Supplement: Supplementary file 1 — Figure S1. Thin layer chromatograph (TLC) of aflatoxins from culture medium extracts. Culture medium from each isolate were filtered and saved for use in aflatoxin quantification. Following extraction, samples from each isolate and treatment were developed with a TLC. Aflatoxins B1 and B2 can be observed on the TLC. Treatments are indicated below for each isolate, stress condition, and timepoint (DAI: days after inoculation). Fluorescence of the aflatoxins is visualized here using ultraviolet light (365 nm). Figure S2. Metabolomics experiment design. Two isolates of Aspergillus flavus, AF13 (highly aflatoxigenic and oxidative stress tolerant) and NRRL3357 (moderate to highly aflatoxigenic and moderately oxidative stress tolerant), were grown in yeast extract sucrose (YES) medium supplemented with either 0 or 15 mM H2O2. Samples were collected at 4 and 7 days after inoculation (DAI). Five biological replicates (n = 5, N = 40) were performed for each isolate, treatment, and time point combination. Statistical comparisons are indicated by the colored arrows with blue indicating oxidative stress effect comparisons, red indicating time effects, and green indicating isolate/genotype effects. (DOCX 842 kb) [file 12866_2019_1580_MOESM1_ESM.docx]

**Responses of the *Aspergillus flavus* metabolome to drought-related oxidative stress are time and isolate dependent.**

**Short Title: *Aspergillus flavus* metabolic responses to oxidative stress**

**Supplemental Information**

Jake C. Fountain^1,2^, Liming Yang^2,3^, Manish K. Pandey^4^, Prasad Bajaj^4^, Danny Alexander^5^, Sixue Chen^6^, Robert C. Kemerait^2^, Rajeev K. Varshney^4^, Baozhu Guo^1,*^

^1^ USDA-ARS, Crop Protection and Management Research Unit, Tifton, GA 31793, USA;

^2^ Department of Plant Pathology, University of Georgia, Tifton, GA 31793, USA;

^3^ Nanjing Forestry University, College of Biology and Environmental Science, Nanjing 210037, China;

^4^ International Crop Research Institute for the Semi-Arid Tropics (ICRISAT), Patancheru, Telangana, India

^5^ Metabolon, Inc., Durham, NC 27713, USA;

^6^ University of Florida, Department of Biology, Genetics Institute, and Plant Molecular & Cellular Biology Program, Gainesville 32611, FL, USA.

Author e-mail addresses: J.C. Fountain: [jfount1@uga.edu](mailto:jfount1@uga.edu); L. Yang: [yanglm@uga.edu](mailto:yanglm@uga.edu); M. Pandey: [m.pandey@cgiar.org](mailto:m.pandey@cgiar.org); P. Bajaj: p.[bajaj@cgiar.org](mailto:bajaj@cgiar.org); D. Alexander: [dalexander@metabolon.com](mailto:dalexander@metabolon.com); S. Chen: [schen@ufl.edu](mailto:schen@ufl.edu); R.C. Kemerait: [kemerait@uga.edu](mailto:kemerait@uga.edu); R.K. Varshney: [r.k.varshney@cgiar.org](mailto:r.k.varshney@cgiar.org); B. Guo: [baozhu.guo@ars.usda.gov](mailto:baozhu.guo@ars.usda.gov)

***Corresponding Author:**

Dr. Baozhu Guo

[baozhu.guo@ars.usda.gov](mailto:baozhu.guo@ars.usda.gov)

**Key Words:** *Aspergillus flavus*, aflatoxin, drought stress, oxidative stress, metabolomics


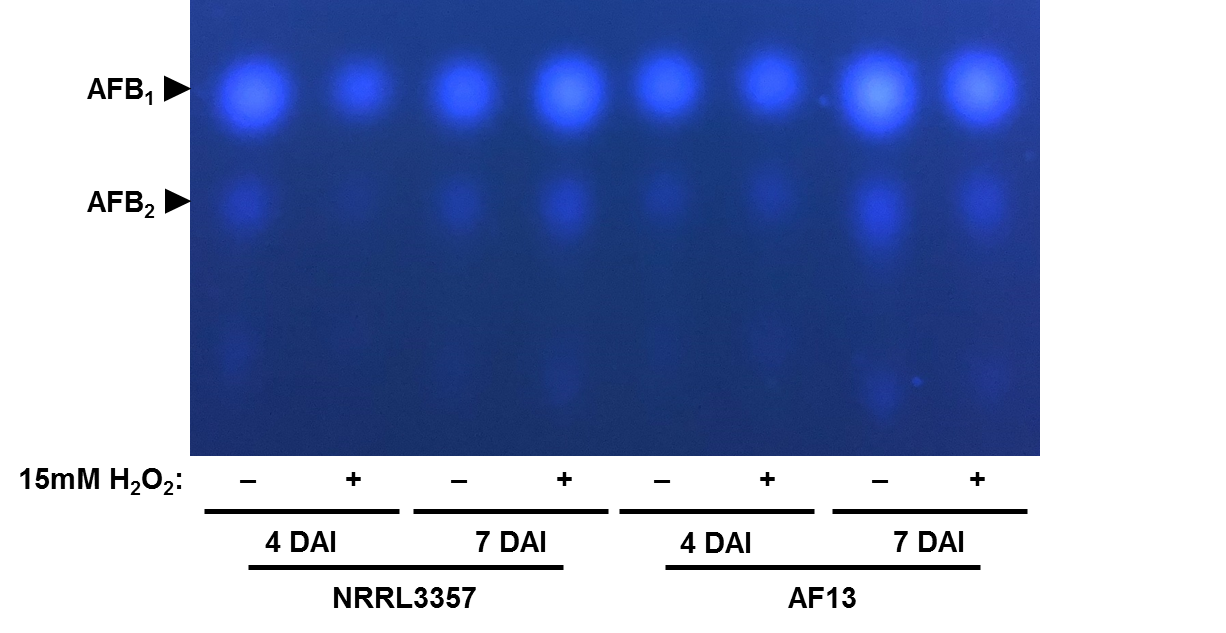


**Figure S1.** Thin layer chromatograph (TLC) of aflatoxins from culture medium extracts. Culture medium from each isolate were filtered and saved for use in aflatoxin quantification. Following extraction, samples from each isolate and treatment were developed with a TLC. Aflatoxins B_1_ and B_2_ can be observed on the TLC. Treatments are indicated below for each isolate, stress condition, and timepoint (DAI: days after inoculation). Fluorescence of the aflatoxins is visualized here using ultraviolet light (365nm).

**
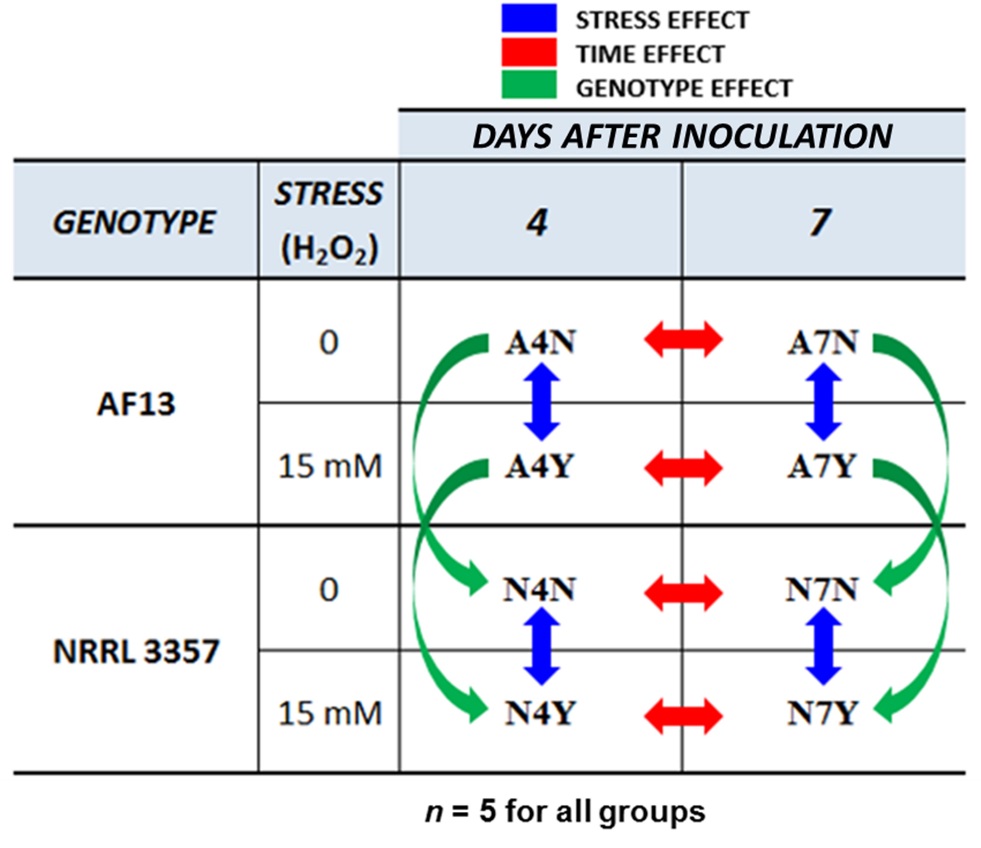
**

**Figure S2.** Metabolomics experiment design. Two isolates of *Aspergillus flavus*, AF13 (highly aflatoxigenic and oxidative stress tolerant) and NRRL3357 (moderate to highly aflatoxigenic and moderately oxidative stress tolerant), were grown in yeast extract sucrose (YES) medium supplemented with either 0 or 15 mM H_2_O_2_. Samples were collected at 4 and 7 days after inoculation (DAI). Five biological replicates (n = 5, N = 40) were performed for each isolate, treatment, and time point combination. Statistical comparisons are indicated by the colored arrows with blue indicating oxidative stress effect comparisons, red indicating time effects, and green indicating isolate/genotype effects.
